# Unusually Aggressive Myofibromatosis In A Neonate

**Published:** 2012-04-01

**Authors:** Jitendra Hazarey, Shilpa Hazare, Girish Moghe

**Affiliations:** Department of Pediatric surgery, Getwell Hospital and Research Institute, 20/1, Dhantoli, Nagpur, India. 440012; 1 Department of Pediatrics, Getwell Hospital and Research Institute, 20/1, Dhantoli, Nagpur, India. 440012; 2 Department of Pathology, Getwell Hospital and Research Institute, 20/1, Dhantoli, Nagpur, India. 440012

A 15 days old male newborn presented with four exophytic swellings arising from the skin of right lower limb; not restricting its mobility (Fig.1). The lesions were sequentially excised and pathological examination revealed infantile myofibromatosis in these growths. No evidence of malignancy was found (Fig.2). The swelling in the thigh recurred after 6 months and suspecting sarcomatous change, a biopsy was done which confirmed the sarcomatous change. Amputation and chemotherapy was offered, and the poor response to chemo-radiotherapy was explained. Parents refused further treatment and child succumbed to the disease after developing inguinal nodes and visceral metastasis.

**Figure:**
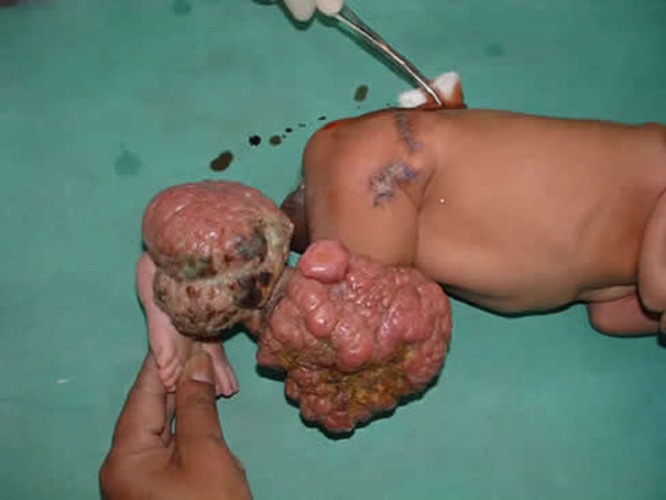
Figure 1: Exophytic masses arising from the knee and ankle. Scar of two excised masses at the hip joint area.

**Figure:**
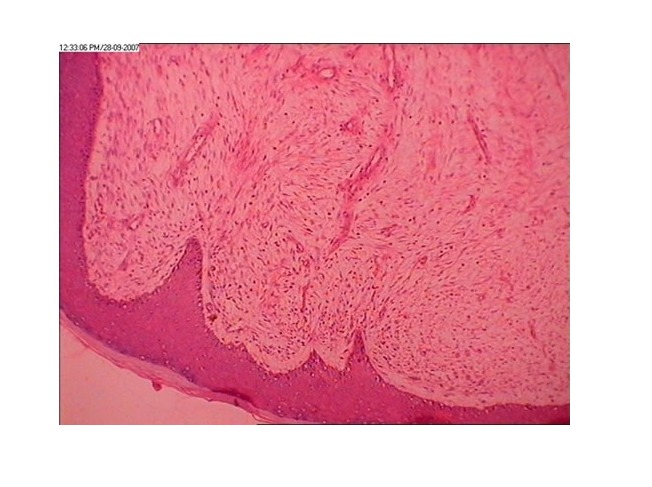
Figure 2: Photomicrograph showing abundant fibroblasts with skeletal muscles. No evidence of malignancy at this time.

Infantile myofibromatosis is a rare tumor of infancy. It is mesenchymal in origin and involves superficial structures or may be visceral. The lesions have been found in nearly all kinds of tissues, including the orbit, bone, lip, oral cavity, central nervous system, gastrointestinal tract, lungs, myocardium, liver, and biliary tree. It may present at birth or appears during the first year of life. The exact etiology is unknown and most cases have been reported as sporadic. Both, autosomal dominant and recessive inheritance modes of transmission have been suggested. The prognosis is excellent in solitary or multicentric lesions without visceral involvement, with possibility of spontaneous regression of lesions confined to the skin, soft tissue and bone, and a very low recurrence rate after surgical excision. The prognosis is poor with visceral involvement [1-3]. In our patient the lesion recurred with a sarcomatous change which is an unusual behavior for a lesion known to be benign with a capacity for spontaneous regression.

## Footnotes

**Source of Support:** Nil

**Conflict of Interest:** None declared

